# Scope of Nursing Practice as Perceived by Nurses Working in Saudi Arabia

**DOI:** 10.3390/ijerph19074220

**Published:** 2022-04-01

**Authors:** Khalid A. Aljohani, Majed S. Alamri, Reem AL-Dossary, Hamdan Albaqawi, Khaled Al Hosis, Mohammed S. Aljohani, Noura Almadani, Bader Alrasheadi, Rawaih Falatah, Joseph Almazan, Jalal Alharbi

**Affiliations:** 1 Department of Community Health Nursing, College of Nursing, Taibah University, Al-Madinah Al-Munawarah 42356, Saudi Arabia; 2 Nursing Department, College of Applied Medical Sciences, University of Hafr Albatin, Hafr Albatin 39524, Saudi Arabia; m.alamri@uhb.edu.sa (M.S.A.); jalaln@uhb.edu.sa (J.A.); 3 Nursing Education Department, Nursing College, Imam Abdulrahman Bin Faisal University, Dammam 34221, Saudi Arabia; rnaldosari@iau.edu.sa; 4 College of Nursing, University of Hail, Hail 81491, Saudi Arabia; h.albaqawi@uoh.edu.sa; 5 Department of Nursing Education, Nursing College, Qassim University, Buraidah 51452, Saudi Arabia; 3747@qu.edu.sa; 6 Department of Medical Surgical Nursing, College of Nursing, Taibah University, Al-Madinah Al-Munawarah 42356, Saudi Arabia; msejohani@taibahu.edu.sa; 7 Community Health Nursing Department, College of Nursing, Princess Nourah Bint Abdulrahman University, Riyadh 84428, Saudi Arabia; naalmadani@pnu.edu.sa; 8 Nursing Department, College of Applied Medical Sciences, Majmaah University, Majmaah 15341, Saudi Arabia; b.alrasheadi@mu.edu.sa; 9 Nursing Administration & Education Department, College of Nursing, King Saud University, Riyadh 11362, Saudi Arabia; rfalatah@ksu.edu.sa; 10 School of Medicine, Nazarbayev University, Nursultan 010000, Kazakhstan; joalmazan030@gmail.com

**Keywords:** scope of nursing practice, nursing needs, nurses, ministry of health, Saudi Arabia

## Abstract

The absence of scope of practice guidelines may lead to role ambiguity and legal consequences in nursing practice. This study measures the scope of practice of nurses in Saudi Arabia. The study utilized a descriptive cross-sectional design using an electronic version of the Arabic Actual Scope of Nursing Practice (A-ASCOP) questionnaire among 928 nurses. Descriptive analysis was followed by a *t*-test and an analysis of variance (ANOVA). Significance was assured through the Bonferroni test; the effect size was measured through partial η2 when appropriate. The A-ASCOP mean score of each dimension ranged from 4.29 to 4.72 (overall mean = 4.59). Significant overall ASCOP score variations were evident, with higher ASCOP among expatriate nurses, females, Hospital Operation Program (HOP) nurses, and nurses with postgraduate qualifications. Partial η2 showed a small effect of <0.016. Low-complexity nursing tasks showed insignificant differences no matter the nurse’s position, but were less practiced by Bachelor of Science in Nursing (BSN) and advanced-degree nurses than by those with a diploma education. High complexity of ASCOP was practiced significantly more often by postgraduate-prepared nurses than by diploma-educated nurses. The study showed that there is a range of variation in nursing practice, but that the lack of internal regulations (nursing scope of practice) has no effect on nursing duties. In a country such as Saudi Arabia, where massive national improvement initiatives are frequent, clearly defining the scope of practice for nurses is essential and needs to be done through government mandates. Further studies are essential to define what the scope of practice should include.

## 1. Introduction

The scope of nursing practice refers to professional nursing activities as defined by state or government law [1]. The optimal scope of practice represents competencies nurses are qualified to perform that are in line with their nursing certification level and meet patients’ needs. In some cases, patients’ needs are beyond a nurse’s scope of practice—either requiring too much or too little of the nurse [2]. Hence, nurses should be skilled in assessing patients and the level of concern in order to deliver a good scope of nursing practice. Quality and safe nursing care is essential in the clinical environment. The contribution of registered nurses (RNs), administrators, educators, and researchers to creating scope of practice guidelines can strengthen and support direct care providers’ practice and help practice adapt to rapidly changing care situations [3,4]. Therefore, the clinical practice setting should develop and communicate authorized policies to provide guidance for safe and evidence-based practice. Nonadherence to the nursing scope of practice leads to poor patient outcomes. Studies show nurses vary in their level of practice; some do not exhibit the full range of competencies, while others may perform irrelevant tasks in response to the needs of the workplace. This calls for a continuous process of reconceptualizing the scope of practice [5,6].

Internationally, nursing practice is driven by professional bodies and influenced by guidelines, codes, and standards [7]. Registered nurses’ roles are impacted by education, nursing processes, collegiality, ethics, collaboration, research, quality, quality of practice, professional practice evaluations, resource utilization, leadership, and communication [1,8]. These international concepts of nursing practice offer diverse definitions of the RN scope of practice yet present a unified endorsement of the professional nurse’s role. The Saudi Commission for Health Specialists (SCFHS) has been the regulatory body for nurse classification and registration since 1992 [5]. Their guidelines focus on qualifications, years of practice, the number of continuing education hours, and international registration for overseas licensed nurses. Although the guidelines have considered different categories of nurses, they have not defined scope of practice within these legal categorizations. The literature reveals few studies highlighting the Saudi RN’s role in different clinical settings and practice situations [5,9].

Aldossary explored both healthcare team and patient perception about nurses’ roles in the Eastern province of Saudi Arabia in detail [5]. The study included 1066 participants who responded to a self-administered questionnaire. Domains such as physical care, professional aspects, and care management were considered the main aspects of a nurse’s role, while psychosocial matters and communication were not identified as aspects of patient care. The identified limitation in the understanding of nurse’s roles, and thus their contributions to patients’ care, works against the national growth of the nursing workforce [9]. 

Therefore, due to the scarcity of Saudi studies defining the nursing scope of practice, further studies are needed to pave the way for future professional development and organizational initiatives to improve patient outcomes and enhance nursing workforce utilization for all levels of nursing, but especially advanced nursing [10,11,12]. The aim of this study was twofold: First, to respond to the urgent need for a scope of nursing practice in the Saudi public healthcare system, and specifically to measure how RNs actually practice in Saudi Arabia. Second, to identify those activities mostly practiced by nurses with different educational backgrounds and positions. This study could inform policy makers, decision makers, and nursing education bodies on how to work toward enhancing nursing workforce achievements.

## 2. Methods

### 2.1. Design

The study utilized a descriptive cross-sectional design that aimed to measure the scope of practice as perceived by nurses working in Saudi Arabia.

### 2.2. Setting and Participants

The study included public hospitals and Ministry of Health (MOH) health centers with a bed capacity of >200 in the western region of the Kingdom of Saudi Arabia. Full-time nurses with six months or more of experience were included in the study. Private hospitals, non-MOH healthcare organizations, and areas other than the western region were excluded.

Therefore, invitations were sent to the targeted study locations, with the aim of attaining 1000 nurses’ responses. Overall, the research team received 1149 electronic responses. However, only 928 responses were eligible for data analysis. Further details are in the results section.

### 2.3. Instrument

The study utilized an electronic version of the Arabic Actual Scope of Nursing Practice (A-ASCOP) questionnaire [13]. Permission to utilize the instrument was granted by the original author as well as the Arabic translation study author [13]. The first part of the study instrument includes sociodemographic data. The second part has 26 items to assess the scope of practice in six dimensions relevant to nursing-related activities. These dimensions are: (1) assessment and care planning, (2) teaching of patients and families, (3) communication and care coordination, (4) integration and supervision of staff, (5) quality of care and patient safety, and (6) knowledge updating and utilization. Reponses were recorded on a 6-point scale (1 = never; 2 = very rarely; 3 = sometimes; 4 = frequently; 5 = almost always; 6 = always). Furthermore, the questionnaire was designed to correspond to different levels of complexity for scope of practice activities. These complexities ranged from level 1 to level 3, where level 1 indicated low complexity, level 2, moderate, and level 3, a high level of complexity. Across the six domains, questions 4, 7, 10, 16, 17, 21, and 25 were related to activities of level 1 complexity. Questions 1, 2, 5, 9, 12, 18, 19, 20, 24, and 26 corresponded to activities of level 2 complexity, while questions 3, 6, 8, 11, 13, 14, 15, 22, and 23 corresponded to activities that could be classified as of level 3 complexity [14]. The first application of the A-ASCOP questionnaire in Arabic was in Lebanon, involving 2307 nurses. The 26 items of the A-ASCOP showed good reliability, Cronbach alpha = 0.93, which indicated that the questionnaire was reliable among Arabic-speaking populations (13).

### 2.4. Data Collection

An electronic version of the A-ASCOP, including an invitation letter, was created on the Survey Monkey website. The invitation information and electronic link were sent to nursing administration departments in public hospitals of the western region, who then forwarded the invitation to their nursing staff. A non-random purposive sampling technique was the applicable approach to reach frontline nurses. Data were collected from 12 July to 27 August 2019.

### 2.5. Data Analysis

Data were analyzed using SPSS (Statistical Package for Social Sciences) version 24. A descriptive analysis of participants’ characteristics was gathered from frequencies, percentages, means, and standard deviation outcomes. To test the ability of A-ASCOP to distinguish actual scope of practice dimensions and the level of complexity among participants’ characteristics, a *t*-test and ANOVA were utilized when appropriate. The homogeneity of variance was checked. The multiple comparison ‘post hoc test’ was applied utilizing the Bonferroni method, which is more rigorous as a conservative method to prevent type I error (α inflation) in such multiple comparisons [15]. The effect size was measured through partial η2 [16]. 

The presentation of data went through two stages. First, participants’ characteristics and corresponding overall A-ASCOP score for each group were identified. Second, earlier studies exploring ASCOP scale dimensions’ means, standard deviations, and their correlations with participants’ education level and current nursing position were followed [16,17].

### 2.6. Ethical Considerations

Participants’ responses remained anonymous and confidential since no identifiable information was collected and all participation was voluntary through online media. Answering the questionnaire was considered consent and acceptance to take part in the study.

## 3. Results

The survey registry recorded 1149 responses. However, after removing incomplete questionnaire responses and those from military and university hospitals, 928 surveys were included in the analysis, with a response rate of 81%. Participants’ characteristics (Table 1) show the majority of the sample were female (82%), hospital nurses (78%), and Saudi nationals (73%). In this study, 98% were directly employed by the MOH or through the Hospital Operation Programs (HOP) contracting system, while the rest were under locum contracting systems. Registered nurses (BSN) accounted for 54% of the sample, while nurse technicians (diploma) represented 41%. The majority were clinical bedside nurses (68%) and in the middle age range (25–44 years old, 90%).

Table 1 showed significant overall ASCOP score variations among socio-demographic characteristics. Expatriate nurses were practicing at the highest ASCOP level (M = 5.10, SD = 0.75) compared to national nurses (M = 4.40, SD = 1.13), t(656) = −10.8, *p* < 0.000. Significant variation was evident in female nurses, who had higher ASCOP scores (M = 4.68, SD = 1.01) than male nurses (M = 4.17, SD = 1.31), t(217) = 4.83, *p* < 0.000. Interestingly, HOP nurses had higher ASCOP levels (M = 4.80, SD = 1.02) than MOH and locum nurses (M = 4.53, SD = 1.10), (M = 4.14, SD = 1.17), respectively; however, a significant difference was evident between MOH and HOP nurses only, F(2925) = 7.41, *p* = 0.001. Similarly, nurses with postgraduate qualifications had higher A-ASCOP levels (M = 4.94, SD = 1.09) than nurses with a diploma education (M = 4.47, SD = 1.16), F(2925) = 5.82, *p* = 0.003. Measuring the partial η2 showed a small effect of <0.016.

As shown in Table 2, the overall mean score for analysis of the study dimensions ranged from 4.29 to 4.72 (overall mean = 4.59). The study sample revealed the most common activities shared by RNs were communication and care coordination (mean = 4.72), while the integration and supervision of staff (mean = 4.29) were less likely activities. Exploring the effect of education and current nurse position revealed that communication and care coordination were more common activities for nurses with postgraduate qualifications (M = 5.08, 1.19) than for those with diploma (M = 4.60, SD = 1.32) or bachelor’s degree qualifications (M = 4.76, SD = 1.17). There was a significant difference between postgraduate and diploma-educated nurses, F(2925) = 4.08; *p* = 0.017. Charge nurses practiced communication and care coordination (M = 4.90, SD = 1.05) more than staff nurses or nurse administrators.

The ASCOP level of complexity practice among study participants (Table 3) showed interesting outcomes where low-complexity tasks were not significantly different in terms of nurses’ position, F(2925) = 1.43; *p* = 0.23. Moreover, low-complexity tasks were more often practiced by BSN and postgraduate nurses than by diploma-educated RNs, F(2925) = 5.86; *p* = 0.003. On the other hand, high-complexity ASCOP was significantly different between postgraduate-prepared nurses and diploma-educated nurses F(2925) = 4.67; *p* = 0.010. High-complexity ASCOP was practiced more by charge nurses 4.65 (1.13), F(2925) = 12.36; *p* ≤ 0.000.

## 4. Discussion

This study provides a first glimpse into the actual scope of nursing practice in Saudi MOH organizations. The findings revealed moderate to high practice levels (M = 4.59, SD = 1.32), indicating higher results than international studies in Lebanon (M = 4.42) and the U.S. (M = 3.21) [16,17]. The findings may reflect the absence of responsibility boundaries between nurses regardless of their education or certification level [5]. Specifically, they demonstrate that nurses with diploma qualifications (40% of the sample) are practicing at advanced nursing levels that they are not prepared for and are possibly risking patient safety [18,19]. 

ASCOP scores were higher among nurses with postgraduate qualifications, followed by those with bachelor’s degrees; nurses with diploma qualifications had the lowest scores. Similar to an earlier national study, advanced education was positively correlated with higher ASCOP [9]. The results agree with an earlier international study showing BSN nurses had broader ASCOP than diploma-educated ones [16]. However, the significance of this study was evident in the differing outcomes between postgraduate and diploma-educated nurses, raising questions about utilizing the nursing workforce in the best interests of patient outcomes [5,20].

Interestingly, these results support earlier international findings that nurse position and role description within the healthcare team has a significant effect towards broadening their ASCOP [16,17,18,19,20,21]. Despite significant findings that the effect supporting participants’ characteristics have on the broadness of ASCOP, measuring the partial η2 showed a small effect of <0.016 [15]. There was no strong evidence that study participants had wide differences in ASCOP in their daily practice, given the above-mentioned characteristics.

In other words, findings such as those of expatriate nurses, who were practicing at higher ASCOP levels than national nurses, and HOP nurses, who had higher ASCOP levels than those in the MOH, have little effect on daily practice. These differences could be attributed to numerous factors including hospital policy and procedures, accreditation level, HOP, and hospital healthcare level, such as tertiary medical centers [22,23,24]. 

The second aim of the study was to identify those activities mostly practiced by nurses with different educational backgrounds and positions. The study revealed that nurses with postgraduate qualifications were practicing broader ASCOP than other nurses in all ASCOP dimensions. However, when it came to nurse position, charge nurses were practicing broader ASCOP, except in the teaching of patients and families. These results could be explained by the fact that those nurses with postgraduate qualifications, such as a Master of Nursing or an advanced nursing practice diploma, most likely work as charge nurses; therefore, the postgraduate characteristics meet the charge nurse position requirements [6]. 

This study revealed that the most common activities performed were communication and care coordination, contrary to earlier international studies, such as in the U.S., where assessment and care planning was widely performed, and in Lebanon, where the teaching of patients and families was most often done [16,17]. On the other hand, in this study and in earlier international ones, the least-reported ASCOP dimension was the integration and supervision of staff. Taking into account that most of the study sample was staff nurses (68%), the integration and supervision of staff may not have been included in their everyday practice [21]. This study confirms earlier findings of international studies where diploma-educated nurses and “nurse assistants” practiced moderate- and high-complexity nursing competencies [17,25,26]. In those studies, high-complexity ASCOP activities were practiced more often by postgraduate as well as charge nurses (13).

### Study Limitations

This study utilized a cross-sectional design, which has inherent limitations in terms of causality or analyzing the ASCOP changes over time. The study did not involve all regions of Saudi Arabia; therefore, the results may not allow for generalization, and further research should be undertaken to get a clear picture of the current nursing ASCOP.

## 5. Conclusions

Nursing ASCOP guidelines are important in improving patient outcomes in collaboration with multidisciplinary healthcare teams. This study showed that there is a range of variation in nursing practice, but the lack of internal regulations (nursing scope of practice) has no effect on nursing duties. In a country such as Saudi Arabia where changes and massive national improvement initiatives are executed every day, defining a Saudi nursing scope of practice is a persistent need that must be met by authorized governmental parties. Further studies should shed light on national nursing issues.

## 6. Implications for Nursing Practice

The study’s findings should motivate nursing management in MOH organizations as well as newly developed national medical clusters to define borders/responsibilities between different nursing registration categories. More importantly, it is imperative to establish guidelines that protect patients as well as nurses from current loose practice boundaries. From a nursing education standpoint, nursing colleges should work as change agents to establish actual scope of practice. Incorporating international nursing competencies within nursing programs may enhance graduates’ understanding of variations among nursing registration categories. Furthermore, nursing colleges should develop or implement nursing regulation courses so nursing students are exposed to Saudi Health Law in order to understand how to protect themselves from practicing out of their competency level.

Nursing legislation is the responsibility of the Saudi Commission of Health Specialties and the MOH. These two bodies are best positioned to implement quick changes in nursing policy and legislation to protect patients and professionals. The third party in this formula is the Ministry of Labour and Social Services who has the chance to meet nursing practice needs that have stood for a long time without governmental intervention. This study’s results highlight the absence of nursing legislation and support organizations such as a nursing council or a nursing association.

## Figures and Tables

**Table 1:** Participants’ characteristics and corresponding overall ASCOP score.

Characteristic	n (%)	Overall Mean (SD)	Stat. Test *p* Value
Gender			
Female	757 (81.6)	4.68 (1.01)	t(217) = 4.83; *p* < 0.000
Male	171 (18.4)	4.17 (1.31)	
Workplace			
Hospital	728 (78.4)	4.66 (1.03)	F(2925) = 8.55; *p* < 0.000 *
PHC	154 (16.6)	4.27 (1.23)	
Others	46 (5.0)	4.47 (0.127)	
Nationality			
Saudi	679 (73.2)	4.40 (1.13)	t(656) = −10.8; *p* < 0.000
Expatriate	249 (26.8)	5.10 (0.75)	
Geographic location in KSA			
Western	194 (20.9)	4.60 (1.04)	F(2925) = 0.028; *p* = 0.97 *
Central	682 (73.5)	4.59 (1.09)	
Northern	52 (5.6)	4.57 (1.22)	
Contract			
MOH	669 (72.1)	4.53 (1.10)	F(2925) = 7.41; *p* = 0.001 *
HOP	240 (25.9)	4.80 (1.02)	
Private (Locum)	19 (2.0)	4.14 (1.17)	
Working experience			
6 months to <1 year	247 (26.6)	4.55 (1.06)	F(2925) = 0.264; *p* = 0.768 *
1–5	285 (30.7)	4.59 (1.12)	
≥6	396 (42.7)	4.62 (1.08)	
Educational level			
Nursing diploma	377 (40.6)	4.47 (1.16)	F(2925) = 5.82; *p* = 0.003 *
Bachelor	500 (53.9)	4.65 (1.02)	
Postgraduate	51 (5.5)	4.94 (1.09)	
Current nursing position			
Staff	630 (67.9)	4.51 (1.09)	F(2925) = 6.45; *p* = 0.002 *
Charge nurse	197 (21.2)	4.82 (0.96)	
Admin	101 (10.9)	4.67 (1.21)	
Age			
18–24	32 (3.5)	4.50 (1.01)	F(2925) = 3.06; *p* = 0.047 *
25–44	828 (89.0)	4.57 (1.10)	
≥45	68 (7.5)	4.90 (1.09)	

* Partial η2 < 0.016.

**Table 2:** Mean (SD) score on ASCOP scale dimensions by nurse education and position type (N = 928).

Dimension	Overall M (SD)	Education			Position		
		Diploma M (SD)	BSN M (SD)	Postgraduate M (SD)	Staff M (SD)	Charge Nurse M (SD)	Admin M (SD)
Assessment and care planning	4.71 (1.21)	4.49 (1.3) *	4.84 (1.11)	5.02 (1.11) *	4.70 (1.18)	4.79 (1.12)	4.64 (1.51)
		F(2925) = 10.9; *p* < 0.000			F(2925) = 1.610; *p* = 0.544		
Teaching of patients and families	4.65 (1.37)	4.56 (1.40)	4.68 (1.34)	5.00 (1.31)	4.67 (1.34)	4.67 (1.32)	4.47 (1.64)
		F(2925) = 2.46; *p* = 0.085			F(2925) = 0.99; *p* = 0.370		
Communication and care coordination	4.72 (1.24)	4.60 (1.32) *	4.76 (1.17)	5.08 (1.19) *	4.65 (1.12)	4.90 (1.05)	4.78 (1.43)
		F(2925) = 4.08; *p* = 0.017			F(2925) = 3.33; *p* = 0.036		
Integration and supervision of staff	4.29 (1.49)	4.15 (1.53)	4.35 (1.45)	4.70 (1.51)	4.05 (1.51) *	4.83 (1.23) *	4.70 (1.46) *
		F(2925) = 3.84; *p* = 0.022			F(2925) = 25.8; *p* = 0.000		
Quality of care and patient safety	4.50 (1.38)	4.43 (1.47)	4.52 (1.32)	4.84 (1.16)	4.34 (1.40) *	4.88 (1.24) *	4.76 (1.31)
		F(2925) = 2.08; *p* = 0.124			F(2925) = 13.6; *p* < 0.000		
Knowledge updating and utilization	4.67 (1.26)	4.55 (1.35) *	4.73 (1.20)	5.02 (0.99) *	4.63 (1.28)	4.83 (1.18)	4.67 (1.24)
		F(2925) = 4.17; *p* = 0.016			F(2925) = 1.94; *p* = 0.144		

* Significant Bonferroni test.

**Table 3:** Mean (SD) score on ASCOP complexity subscale dimensions by nurse education and position type (N = 928).

Dimension	M (SD)	Education			Position		
		Diploma	BSN	Postgraduate	Staff	Charge Nurse	Admin
Low complexity	4.83 (1.08)	4.69 (1.19) *	4.92 (0.98) *	5.04 (0.97)	4.81 (1.07)	4.94 (0.98)	4.75 (1.29)
		F(2925) = 5.86; *p* = 0.003			F(2925) = 1.43; *p* = 0.23		
Moderate complexity	4.70 (1.12)	4.57 (1.21) *	4.75 (1.04)	5.09 (0.99) *	4.63 (1.13) *	4.90 (0.97) *	4.72 (1.26)
		F(2925) = 6.04; *p* = 0.002			F(2925) = 0.4.60; *p* = 0.010		
High complexity	4.32 (1.28)	4.20 (1.32) *	4.37 (1.25)	4.74 (1.13) *	4.18 (1.31) *	4.65 (1.13) *	4.56 (1.25) *
		F(2925) = 4.67; *p* = 0.010			F(2925) = 12.36; *p* ≤ 0.000		

* Significant Bonferroni test.

## Data Availability

Not applicable.
